# Amplifying radiation-induced anti-tumor immunity: the dual role of brachytherapy and low-dose total body irradiation

**DOI:** 10.3389/fimmu.2026.1771593

**Published:** 2026-02-17

**Authors:** YingQi Gu, Yang Yang, Jian Gao, ZhouXue Wu, Jia Wang, Chen Xie, XinYi Wang, Min Wu, YunXue Zheng, XiaoYin Zhang, Yue Chen, ShaoZhi Fu, JingBo Wu

**Affiliations:** 1 Department of Oncology, The Affiliated Hospital, Southwest Medical University, Luzhou, Sichuan, China; 2 Emergency Department of the People’s, Hospital in Luding County, Garze Tibetan Autonomous Prefecture, Luzhou, Sichuan, China; 3 Department of Respiratory and Critical Care Medicine, The Affiliated Hospital, Southwest Medical University, Luzhou, Sichuan, China; 4 Department of Tumor Blood Radiotherapy, Luzhou People’s Hospital, Luzhou, Sichuan, China; 5 Nuclear Medicine and Molecular Imaging Key Laboratory of Sichuan Province, Luzhou, Sichuan, China; 6 Academician (Expert) Workstation of Sichuan Province, Luzhou, Sichuan, China

**Keywords:** hypo-fractionated brachytherapy, hypo-fractionated radiotherapy, immunogenic cell death, low-dose total body irradiation, systemic immune related response

## Abstract

**Background:**

Radiotherapy can be a vaccine by triggering patients’ prophylactic tumor-specific immune responses. Brachytherapy has biological and physical benefits over external beam radiation. Low-dose total body irradiation can produce systemic immunity. We hypothesized that brachytherapy more effectively modulates immunity than external beam radiotherapy, with low-dose total body irradiation amplifying this effect.

**Methods:**

After creating the Lewis lung cancer model, we compared hypo-fractionated brachytherapy with hypo-fractionated radiotherapy, examining immunogenic cell death, DNA damage, cell proliferation and immune cells in tumor. We then evaluated if low-dose whole-body irradiation could boost hypo-fractionated brachytherapy’s systemic immunomodulatory effects and trigger a distant response.

**Results:**

Hypo-fractionated brachytherapy was more effective in inhibiting tumor growth than external beam radiotherapy. Hypo-fractionated brachytherapy approach significantly influenced various immune cells within the tumor microenvironment, including T cells, DC cells, NK cells, MDSC cells, tumor-associated macrophages. Furthermore, low-dose total body irradiation at 0.1 Gy augmented the immunological effects of low-fractionation brachytherapy and elicited transient systemic immune activation in mice.

**Conclusions:**

Our research indicates that brachytherapy offers superior immune modulation over external radiotherapy. When combined with low-dose total body irradiation, it transiently activates the systemic immune response.

## Introduction

According to cancer statistics, there will be 19.3 million new cancer cases and nearly 10 million cancer deaths worldwide in 2020. Lung cancer is the leading cause of cancer deaths, followed by colorectal cancer, liver cancer, breast cancer, and stomach cancer (1).

Radiotherapy (RT) has been used for more than 50% of cancer patients. With the use of radioactive elements as a mature treatment modality, brachytherapy (BT), an internal irradiation modality, is increasingly used. Brachytherapy (BT) is considered standard treatment for men with intermediate prostate cancer (2). BT offers significant dose advantages, delivering high doses locally to tumors while minimizing damage to normal tissues (3, 4). With the emergence of CT, MRI, PET and other auxiliary technologies, brachytherapy makes local control, survival rate, and quality of life (QOL) significantly improved, and treatment sequelae reduced (5–7). This makes brachytherapy an effective treatment for localized tumors.

Although radiotherapy can sometimes lead to the regression of distant tumors, this phenomenon remains a rare occurrence in clinical practice. X-ray total body irradiation (TBI) was previously generally considered as a method of suppressing the immune response of organisms and was used to eliminate the immunity of experimental animals and was also used for interdisciplinary treatment of hematopoietic diseases and malignancies (8). Until there were experiments to investigate whether tumor cell transplantation could be completed after TBI, it was found that tumor cell transplantation failed after 10 or 15cGy TBI, and the rejection effect was significant when tumor cells were transplanted between 10 and 15h after TBI, indicating that this low-dose total body irradiation(L-TBI) may change the anti-tumor immune status of the host and promote the immune response of the organism (9). Later, a large amount of evidence showed that exposure to ionizing radiation at low doses can significantly delay tumor development and inhibit distant metastasis of tumor cells in humans and experimental animals. L-TBI can also reduce apoptosis of immune cells such as NK cells and DCs in spleen (10–12). This positions L-TBI as a promising approach to improve systemic immune response (SIME).

In our previous experiments with mouse models of breast and colon cancer, we combined hypo-fractionated radiotherapy (H-RT) with low-dose total body irradiation (L-TBI). This combination potentially enhanced SIME by increasing CD8+ T cell infiltration and modifying the immunosuppressive environment in non-irradiated tumor sites, significantly inhibiting secondary tumor growth through IFN-γ production by tumor-specific CD8+ T cells (13). Building on this foundation, we concentrate on brachytherapy, a unique form of radiation therapy different from external beam radiation. It provides low toxicity and precise dose delivery to the target area, but its immune effects remain largely unexplored. We aim to determine if brachytherapy offers more immunomodulatory benefits than external beam radiotherapy, a topic seldom explored. Additionally, we’ll evaluate if L-TBI can boost brachytherapy’s effects to aid clinical application.

## Materials and methods

### Mice and cells

C57BL/6J mice (female, 6 weeks old,20g) were obtained from Chongqing Tengxin Biotechnology Co., Ltd. (Chongqing, China). In immunological experiments, female mice are preferred due to the significant impact of androgens on immune indicators. All animal experiments were approved by the Animal Care and Ethics Committee of Southwest Medical University (Luzhou). Lewis lung carcinoma (LLC) cell line was purchased from (Cell Bank of the Chinese Academy of Sciences, China) and the cells were cultured in Dulbecco’s modified Eagle medium (L110KJ, BasalMedia) supplemented with 10% fetal bovine serum and 1% penicillin-streptomycin. The cell cultures were incubated in a humidified incubator at 37 °C and 5% CO_2_.

A tumor model was developed by injecting 100ul of 1×10^^6^.LLC cells subcutaneously into a mouse’s right leg. Radiotherapy was administered once the tumor reached 250 mm³, with volume calculated using the formula: length × width × width × 0.5. After ear-tagging the mice, randomization was done using Excel’s random number generator. Each group had 5 mice to adhere to the “reduction” principle of the 3Rs, using the minimum number needed for clear results. The groups were labeled as control, H-BT, H-RT, and L-TBI+H-BT. To minimize bias in quantitative assessments, we ensured objective measurements like animal weight and biochemical indicators were directly generated by instruments, reducing human error. For subjective assessments, particularly histopathology scoring, we enforced blinding by having tissue sections randomly coded by another researcher and evaluated by a pathologist unaware of group assignments.

### L-TBI and H-RT

The mice were fixed in our home-made small animal radiotherapy box, exposing the whole right leg through a small hole, leaving the right leg in a stretched state and the left leg in a natural state. All right legs and primary tumors were placed in the radiation field for radiotherapy. Our radiotherapy box had been tested in an ionization chamber before radiotherapy. Dose rates in the center and middle planes of the irradiation field were measured. In addition, thermoluminescence films were stacked near the tumor to verify the dose. L-TBI or H-RT was delivered with a 6MV linear accelerator (VarianClinac600C, USA) at a source-to-surface distance of 100 cm. L-TBI was whole-body irradiation at a dose rate of 24cGy/min, a dose rate of 0.1 Gy/min, and a primary H-RT of 8Gy×3, a dose rate of 400cGy/min.

### Hypo-fractionated brachytherapy

Mice were anesthetized with 2% isoflurane for induction and 1% isoflurane for maintenance using a small animal anesthesia machine (Reddot R540, China). The needle was inserted into the tumor’s distal edge after taping the mouse’s limbs. The needle was cleaned with an iodophor wipe between applications. The 192Ir high dose rate source was dispensed by a remote afterloader (Shinav, XHDR39) into the needle placed at the tail of the tumor. Brachytherapy prescription was 8 Gy proximal to or cranial to the margin of the tumor. (Depth range 5-10mm, depending on tumor size; dose rate ≥0.12Gy/min, depending on age of 192Ir source). Medical physics staff conducted dose calibration and quality assurance checks on the irradiators as follows:

The radioactive source used in this study is iridium-192 (^192^Ir). The precise half-life of this source is 73.83 days (commonly approximated as 74 days in clinical practice). The detector used to measure the source’s output dose rate and perform equipment radioactivity calibration is a well-type ionization chamber, specifically the HDR 1000 PLUS WELL CHAMBER model. This is the dedicated standard equipment for calibrating high-dose-rate brachytherapy sources. we employ the nationally benchmark-calibrated HDR 1000 PLUS WELL CHAMBER well-type ionization chamber to perform periodic absolute measurements and calibrations of the output dose rate for brachytherapy unit radiation sources, ensuring the accuracy of dose delivery.

All daily quality control, periodic calibration, and radiation physics assurance for radiotherapy equipment (including brachytherapy units and planning systems) are strictly executed and documented by our Medical Physics Department in accordance with relevant national regulations and industry standards. This ensures the safety and precision of clinical treatments.

Supplementary Key Parameters for This Study:

Treatment Equipment: Shandong Xinhua Brachytherapy System, Model XHDR30.

Initial Radioactive Source Activity: 10 Ci.

Treatment Planning System: Shandong Xinhua TPS3d10, employing inverse optimization algorithms for plan design.

Dose Targets: Plans are optimized to ensure 80% of the prescribed dose covers the target volume (tumor mass).

### Flow cytometry

Mice were euthanased by cervical dislocation. Tumor and spleen tissues were taken and placed in DMEM medium, and the tissues were cut and ground respectively with scissors at 4° ice. Tumors were then incubated for 20 minutes at 37 °C in digestive enzymes containing 0.2% collagenase IV, 0.01% hyaluronidase and 0.002% DNase I and spleen was lysed in red blood cell lysate for 15 minutes (Biosharp). The cells were then filtered with filter loops, the single cell suspension thus obtained was used to identify viable cells with FVS780-APC-cy7(BD Pharmingen), the entire immune cell population was labeled with CD45-Pe-cy7(BD Pharmingen), and the remaining surface stained was labeled with the following antibodies: CD3-APC, CD8-PE, CD4-FITC, NK1.1-PE, CD11b-APC, CD11c-PE, CD86-FITC, IA-IE-Percp-Cy5.5, CD11b-FITC, GR-1-APC, F4/80-Percp-Cy5.5 antibodies(BD Pharmingen), according to manufacturer’s protocol After surface labeling with all antibodies, cells were treated with a fixation and permeabilization kit (BD Pharmingen) followed by CD206-PE (Biolegend)staining, then washed twice, and finally stained samples were analyzed using Beckman Coulter Gallios flow cytometry (Beckman Coulter, Miami, Florida, USA).

### Immunohistochemistry

Tumor tissues were fixed in 10% neutral buffered formalin and embedded in paraffin, and sections 4μm thick were cut and used for immunohistochemistry (IHC). Sections were labeled with the following antibodies according to the manufacturer’s instructions: Ki67,γ-H2AX, calreticulin (CALR), (high–mobility group box 1)HMGB1, (.Heat shock protein 70)HSP70. Image-j were taken using a digital slide scanner.

### Elisa

Following deep isoflurane anesthesia, blood was drawn from the mice’s orbital cavity and kept at room temperature for 45 minutes before the mice were euthanized. The blood samples were then centrifuged at 2500 rpm for 20 min to obtain the upper serum layer INF-γ, CXCL10, IL-2, IL-10 levels were measured by a specific antibody ELISA kit according to the manufacturer’s instructions (Abin) by standard ELISA methods.

### Statistics

Statistical analysis was performed using GraphPad Prism V.9.3.1 software. Data are expressed as mean ± SEM. After verifying that the data follows a normal distribution. One-way ANOVA and Tukey’s test compared column means across multiple data sets, while a T-test was used for two data sets, with p-values under 0.05 deemed significant.

## Results

### H-BT inhibited tumor growth more effectively and induced more DNA damage than H-RT

**Figure 1:**
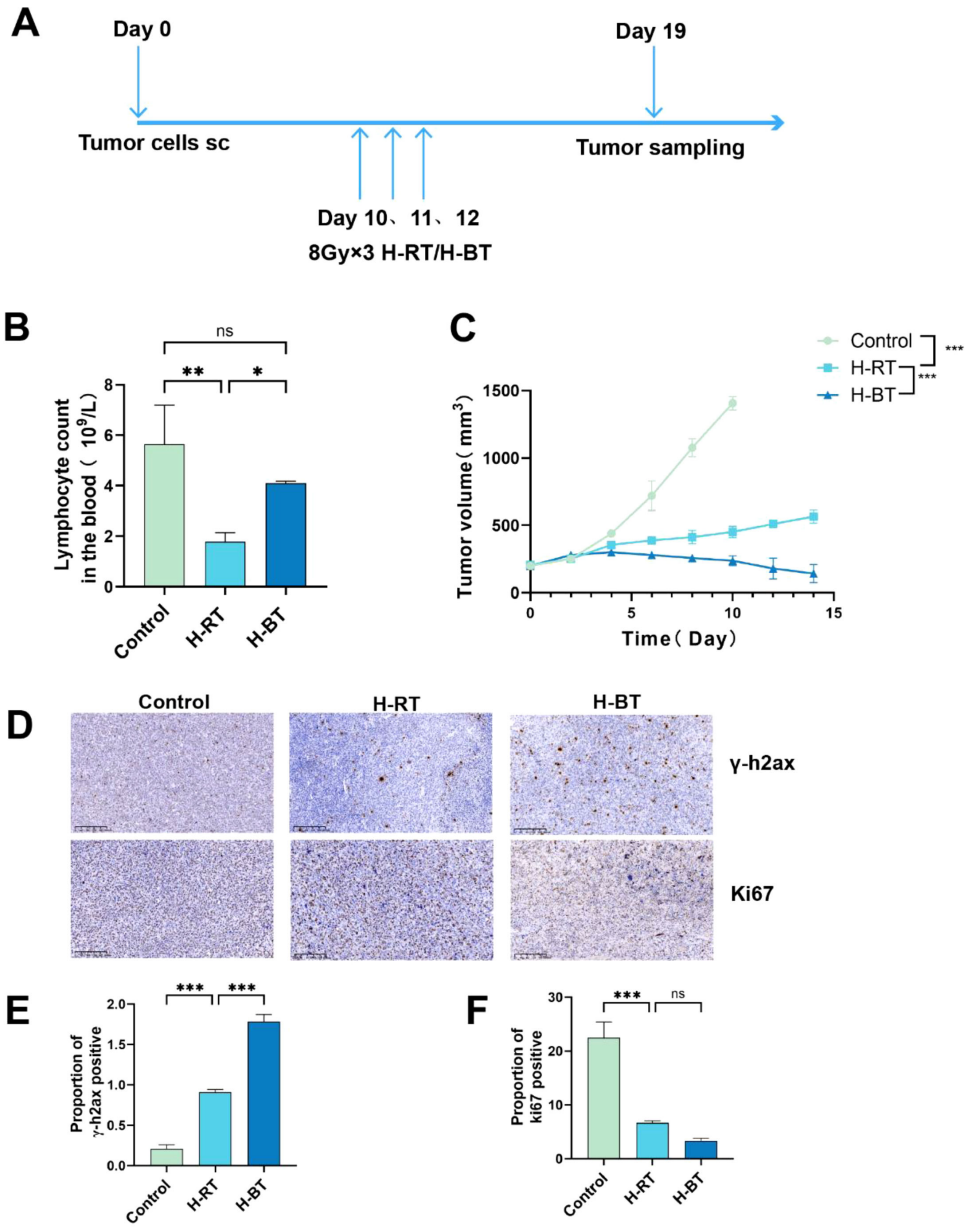
Post-irradiation blood lymphocyte levels, tumor growth trends, and *γ*-H2ax and Ki67 immunohistochemistry. **(A)** Flow chart of radiotherapy. **(B)** Lymphocyte counts in the blood of mice after radiotherapy, H-BT group were significantly higher than H-RT group (P < 0.05). **(C)** Subcutaneous tumor growth curve of LLC model mice, H-BT group was significantly lower than H-RT group (P<0.001).**(D–F)** Immunohistochemistry revealed significantly higher γ-H2AX expression in the H-BT group compared to the H-RT group (P<0.001). *P < 0.05, **P < 0.01, ***P < 0.001.

Radiotherapy was administered to mice using two protocols: 8 Gy x 3 H-BT and H-RT (**Figure 1A**). Hematologic toxicity was evaluated by measuring lymphocyte counts. Lymphocyte counts were significantly lower in the H-RT group compared to the control group whereas in the H-BT group, lymphocyte counts were higher than in the H-RT group (**Figure 1B**). Radiotherapy led to a more pronounced suppression of tumor growth in the H-BT group compared to the H-RT group, accompanied by a concomitant increase in γ-H2AX-positive cells (**Figures 1C–E**).

### H-BT promotes immunogenic cell death more effectively than H-RT

**Figure 2:**
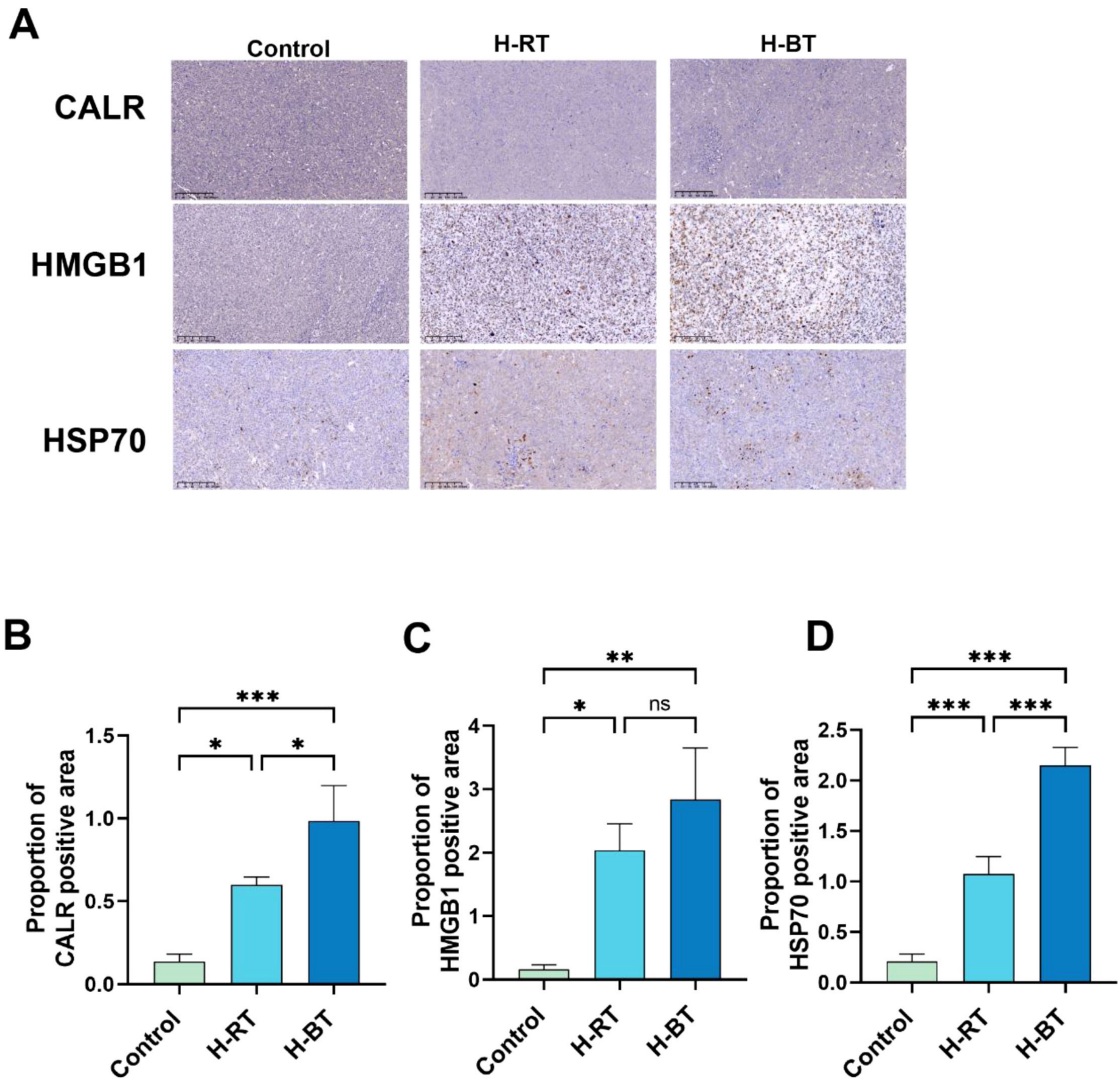
Immunogenic cell death after various radiotherapy treatments. **(A)** Immunohistochemistry of CALR, HMGB1 and HSP70 expression in tumor. **(B)** CALR expression level in H-BT is significantly higher than that H-RT (P<0.05). **(C)** HMGB1 levels were higher in the H-BT group compared to the H-RT group (P>0.05). **(D)** HSP70 expression levels were significantly increased in H-BT compared to the H-BT (P<0.001). *P < 0.05, **P < 0.01, ***P < 0.001.

We assessed whether H-BT and H-RT induce ICD differently (**Figure 2A**). Immunohistochemical analysis showed that H-BT significantly increased CALR and HSP70 expression compared to H-RT, while HMGB1 levels rose without significant difference (**Figures 2B–D**).

### Effect of H-BT on immune cells in tumor site

**Figure 3:**
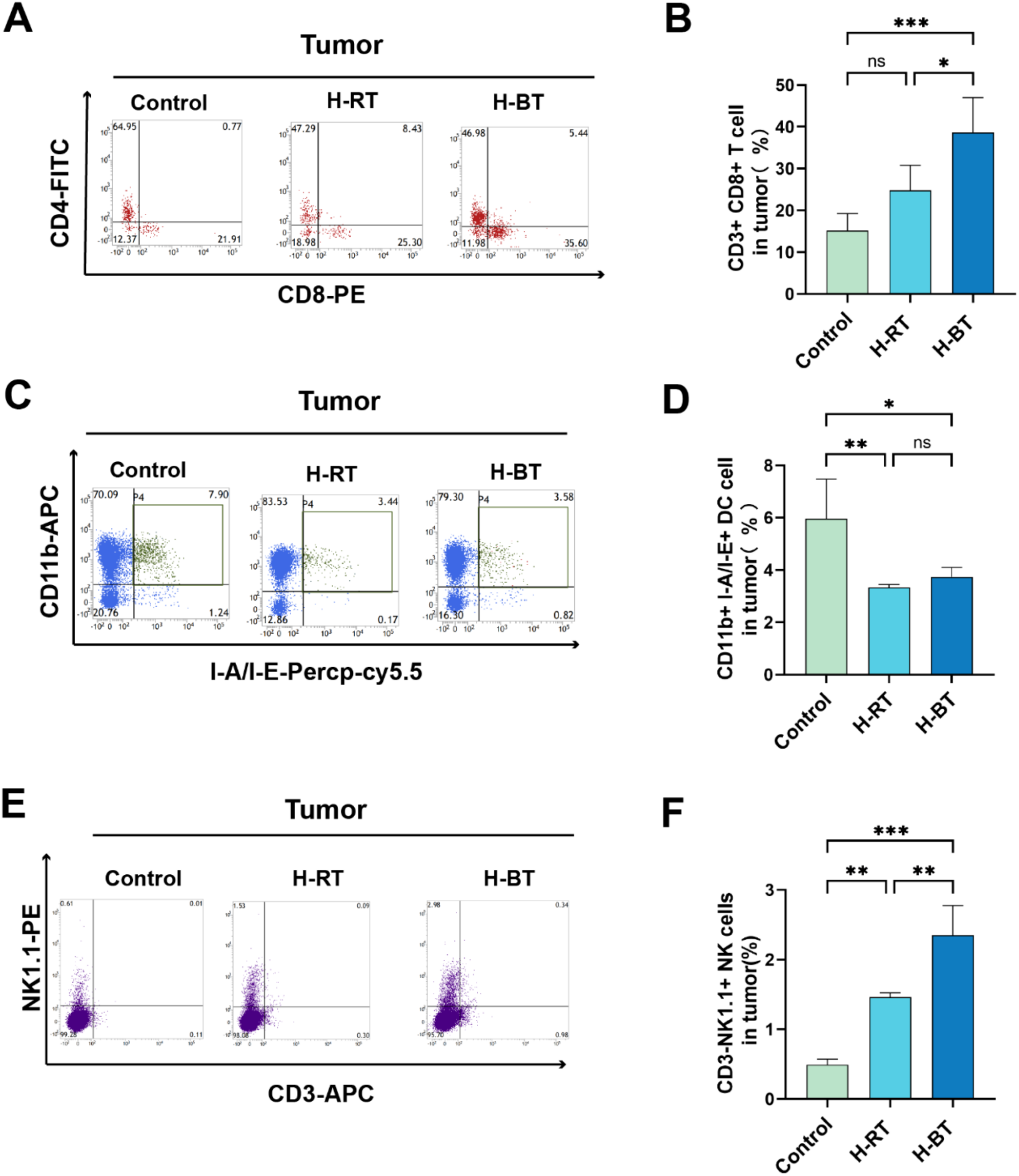
Infiltration of CD8+ T cells, dendritic cells, and natural killer cells at tumor sites after various radiotherapy treatments. **(A, B)** CD8 +T cells in H-BT group significantly higher than that in H-RT group (P<0.05). **(C, D)** No significant differences were observed in DC cells within tumors. **(E, F)** H-BT significantly increases the proportion of NK cells compared with H-RT (P<0.01). *P < 0.05, **P < 0.01, ***P < 0.001.

The infiltration of immune cells into the tumor site was evaluated. A significantly higher percentage of CD8^+^ T cells was observed in the H-BT group compared to the H-RT group (**Figures 3A, B**). Although the H-BT group exhibited a greater number of cDC1 cells, this difference did not reach statistical significance (**Figures 3C, D**). Additionally, the proportion of NK cells in the tumor microenvironment was significantly increased in the H-BT group compared to the H-RT group (**Figures 3E, F**).

**Figure 4:**
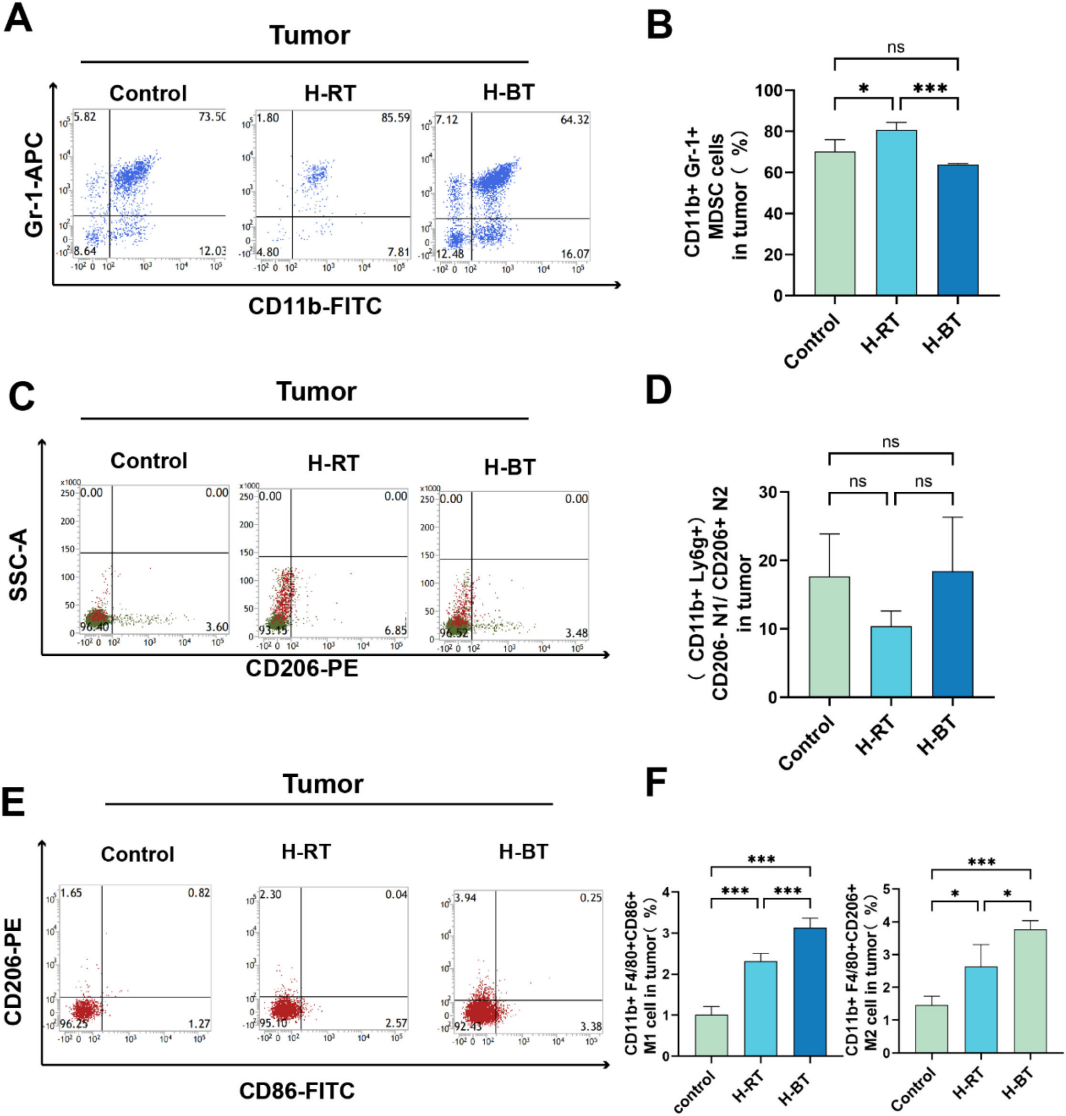
The proportion of MDSCs, tumor-associated macrophages, and neutrophils in tumor tissues after various radiotherapy treatments. **(A, B)** H-BT significantly reduced the proportion of MDSCs (CD11b + Gr-1+) (P<0.001), H-RT alone increased MDSC infiltration (P<0.05). **(C, D)** H-RT and H-BT did not significantly impact tumor-associated neutrophils (CD11b+Ly6g+CD206+/CD206-). **(E, F)** The H-BT group (P < 0.001) significantly promoted the polarization of anti-tumor M1 macrophages (CD11b+F4/80+CD86+) and simultaneously increased M2 (CD11b+F4/80+CD206+) M2-type polarization. *P < 0.05, ***P < 0.001.

The presence of myeloid-derived suppressor cells (MDSCs) at tumor sites following radiotherapy was assessed. The proportion of MDSCs was significantly lower in the H-BT group than in the H-RT group (**Figures 4A, B**). In contrast, the H-RT group exhibited a higher proportion of MDSCs compared to the Control group. Furthermore, analysis of macrophage polarization revealed that the H-BT regimen significantly promoted M1 anti-tumor macrophage polarization (**Figures 4E, F**). Neither H-RT nor H-BT had a significant effect on neutrophil levels (**Figures 4C, D**).

### Effect of L-TBI+H-BT on immune cells of spleen

**Figure 5:**
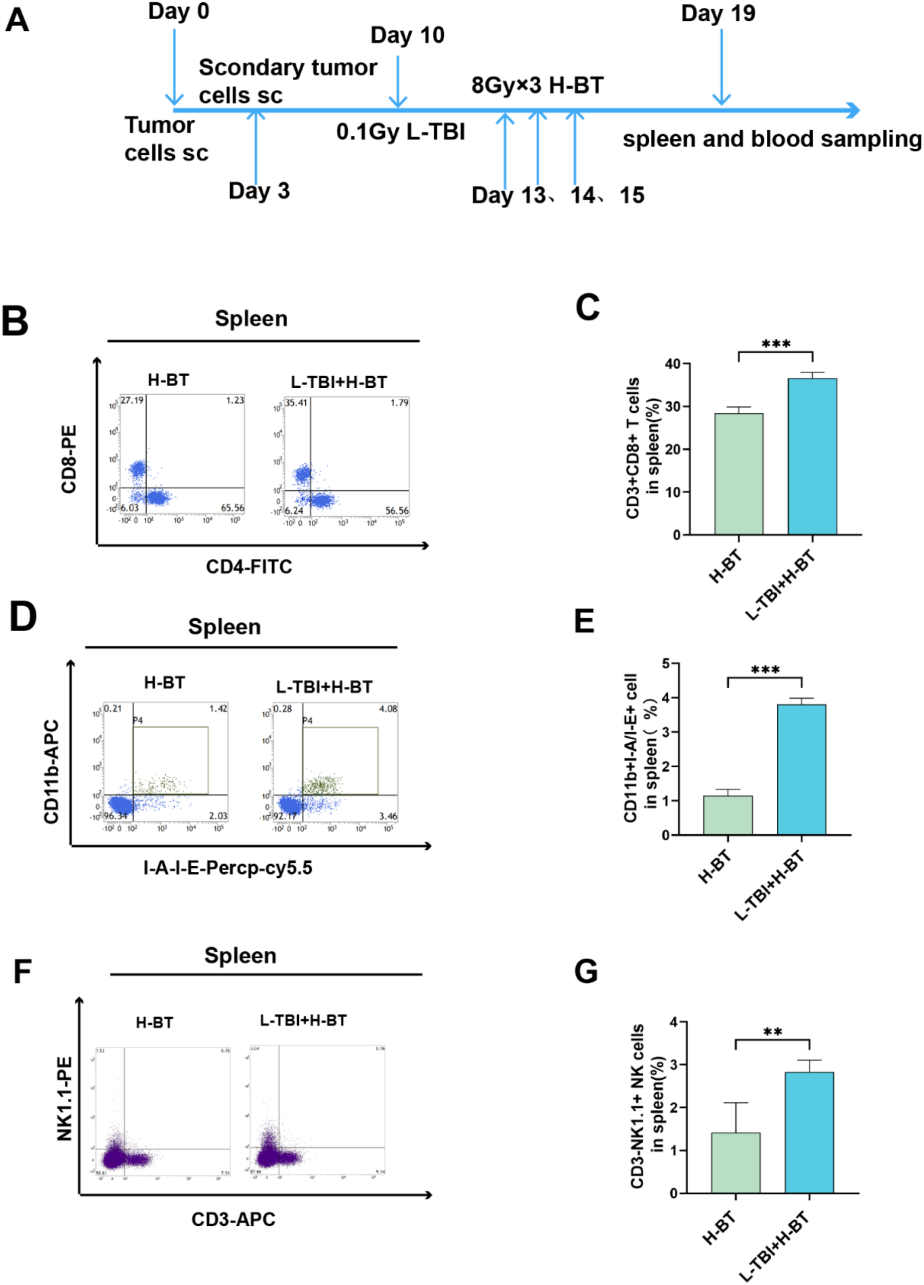
Proliferation of CD8+ T cells, dendritic cells, and natural killer cells in the spleen. **(A)** Bilateral tumor inoculation,H-BT following the L-TBI. **(B,C)** L-TBI+H-BT significantly elevated the proportion of CD8+ T cells (CD3+CD8+) (P<0.001). **(D, E)** L-TBI+H-BT promotes DC (CD11b+IA/IE+) proliferation more effectively (P<0.001). **(F, G)** L-TBI+H-BT can significantly enhance the activation of NK cells(CD3-NK1.1+)(P<0.01). **P < 0.01, ***P < 0.001.

Develop a bilateral tumor model to assess if low-dose total body irradiation (L-TBI) temporarily boosts systemic immune responses. H-BT was delivered to the tumor on the irradiated side three days after L-TBI (**Figure 5A**).

In the spleen, L-TBI+H-BT significantly increased the proportion of CD8^+^ T cells (**Figures 5B, C**) and promoted dendritic cell (DC) proliferation more effectively than H-BT alone (**Figures 5D, E**). NK cell activation was also significantly enhanced by L-TBI+H-BT (**Figures 5F, G**).

**Figure 6:**
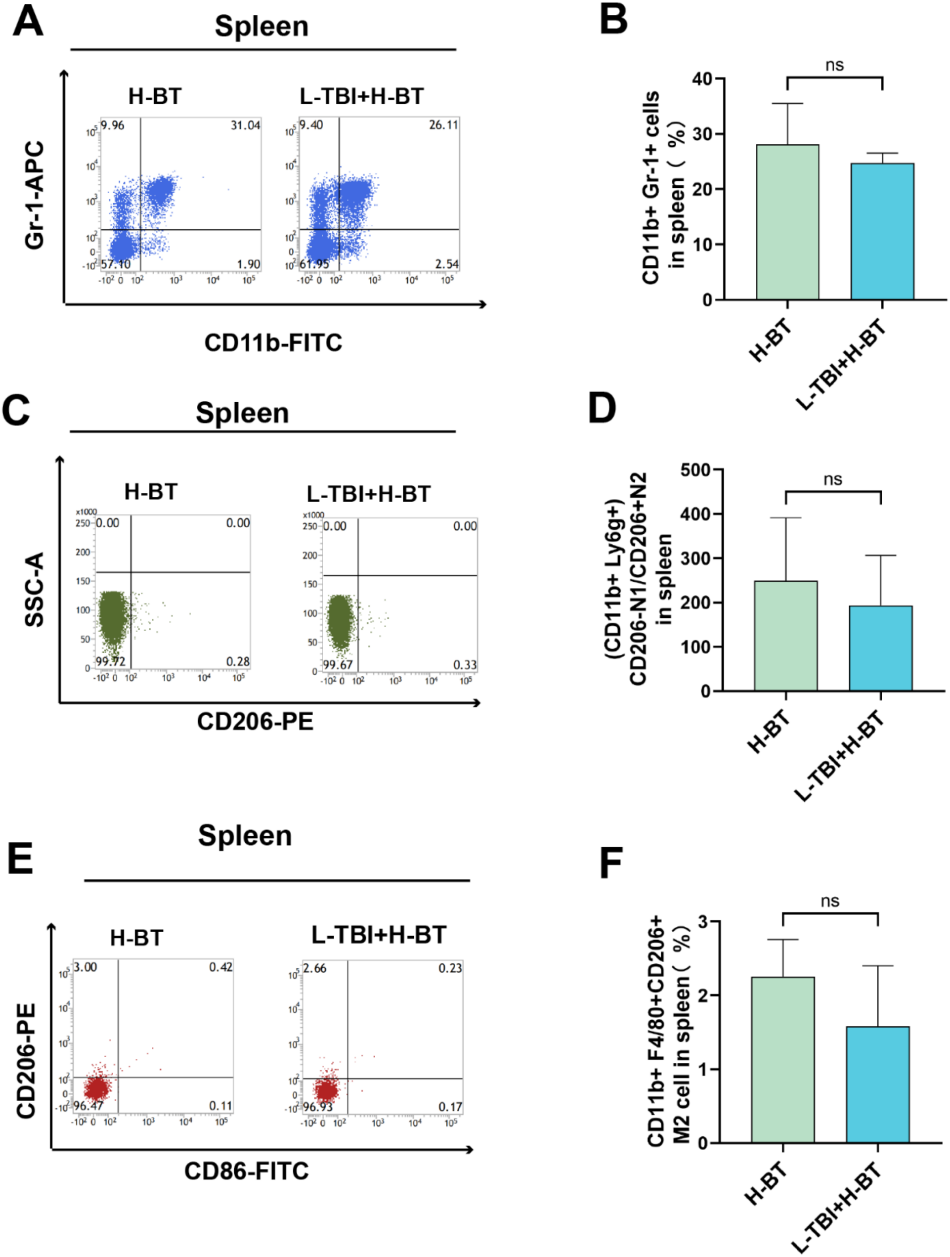
Splenic MDSC, tumor-associated neutrophils and macrophages. **(A, B)** MDSC cells (CD11b+Gr-1+) in spleen **(C, D)** N1/N2 cells (CD11b+Ly6g+CD206+/CD206-) in spleen. **(E, F)** M1 cells (CD11b+F4/80+CD86+) in spleen.

No significant differences between H-BT and L-TBI+H-BT were observed in splenic MDSCs, tumor-associated neutrophils, or M2 macrophages (**Figures 6A–F**).

### L-TBI+H-BT boosted anti-tumor factors CXCL10, IFN-γ, and IL-2, reduced tumor-promoting IL-10, and triggered regression in distant tumors

**Figure 7:**
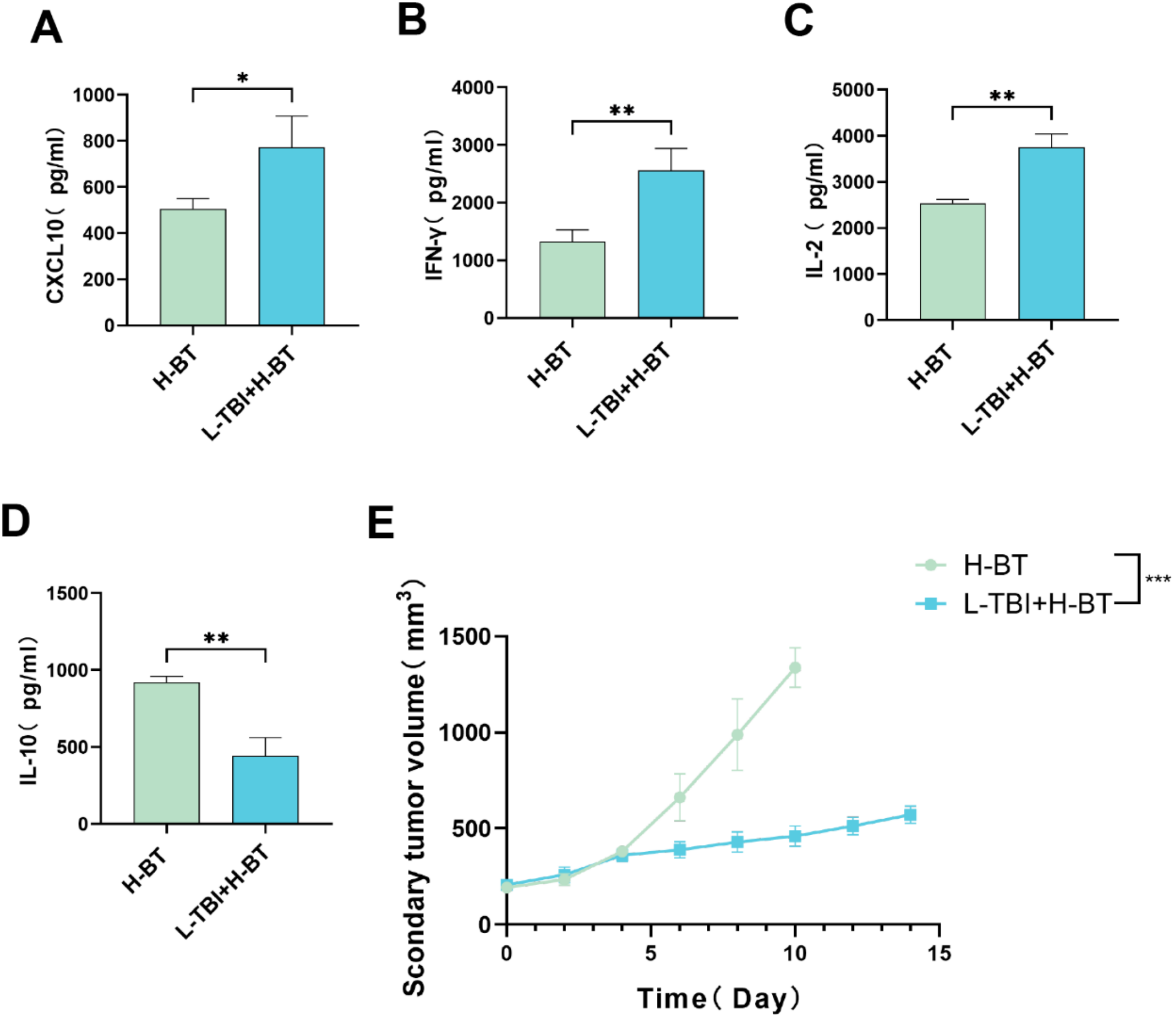
CXCL10, IFN-γ, IL-2, and IL-10 levels in blood and tumor growth on the non-radiated side. **(A)** CXCL10 expression levels were significantly higher in the L-TBI+H-BT group than in the H-BT (P<0.05). **(B)** compared with H-BT, L-TBI+H-BT showed higher IFN-γ level (P<0.01). **(C)** L-TBI+H-BT exhibited a more significant increase in IL-2 levels than H-BT(P<0.01). **(D)** L-TBI+H-BT decreased IL-10 production relative to H-BT. **(E)** The addition of L-TBI delayed tumor growth on the contralateral side. (P<0.01). *P < 0.05, **P < 0.01, ***P < 0.001.

We measured blood levels of tumor-related cytokines. The L-TBI+H-BT group exhibited significantly higher expression of CXCL10 (**Figure 7A**) and IFN-γ (**Figure 7B**) compared to the H-BT group. IL-2 levels were also increased in the L-TBI+H-BT group (**Figure 7C**), whereas IL-10 production was significantly reduced (**Figure 7D**).

We evaluated if adding L-TBI enhanced the temporary systemic immune response by observing the growth of contralateral non-irradiated tumors. The L-TBI+H-BT combination significantly inhibited tumor growth on the untreated side (**Figure 7E**), an effect not observed with H-BT alone.

## Discussion

Our findings show that H-BT causes more DNA damage and immunogenic cell death than H-RT (**Figures 1**, **2**). Additionally, H-BT led to notable immune changes at the tumor site, increasing CD8+ T cells and NK cells, decreasing MDSC cells, and promoting anti-tumor macrophage differentiation (**Figures 3**, **4**). No author has directly compared radiotherapy and external beam radiation therapy like we have, but studies on Carbon Ion Radiation Therapy(CIR) versus photon irradiation show CIR triggers immune responses with increased CD8+ T cells and T effector memory cells in the spleen, enhanced IFN-γ production by CD8+ tumor-infiltrating lymphocytes, and reduced T cell exhaustion in tumors and the spleen. They also noted dose-dependent increases in cytoplasmic dsDNA, dsDNA-activated cGAS-STING pathway proteins (p-TBK1 and p-IRF3), and downstream interferon-stimulated gene expression after CIR. his shows that various radiotherapy methods differently affect immune regulation. Although we compared H-BT with H-RT, it is uncertain what dose or combination is the optimal treatment. Studies have shown that radiation dose may have a critical effect on immune activation. Trex1 is considered to be a key determinant of limited immunogenicity of cancer cells in response to high doses of radiation. Radiation doses higher than 12–18 Gy induce the expression of Trex1, a DNA exonuclease, and degrade DNA accumulated in cytosol during radiation to reduce its immunogenicity (14–16). Further research is needed to determine the optimal radiation therapy dosage regimen.

While radiotherapy has the potential to stimulate immune cell proliferation and exert systemic immunomodulatory effects, its capacity to elicit a robust systemic immune response in clinical settings is relatively constrained. Clinical investigations reveal that stereotactic body radiotherapy (SBRT) alone results in increased CD8+ T cell counts in only a minority of patients and does not significantly enhance long-term survival outcomes. Furthermore, the addition of immune checkpoint inhibitors does not substantially augment this effect. Consequently, relying solely on radiotherapy is inadequate for effectively stimulating systemic antitumor immunity, underscoring its clinical limitations. To achieve synergistic effects, it is imperative to integrate systemic therapies.

Radiation-induced distant tumor regression is known as the distant effect, and rare cases of distant effects in melanoma have been previously reported (17, 18). This may be due to SIME after radiotherapy. But Localized radiotherapy frequently lacks a robust *in situ* effect. A common strategy for enhancing SIME is to combine RT with immunotherapy (19–21). However, L-TBI effectively induces systemic immune responses. Earlier research demonstrated that low-dose whole-body irradiation (L-TBI) at 2 Gy boosts CD4+ T cell-driven antitumor immunity via Th1 polarization (22). Low-dose radiotherapy acts as an “immunostimulant” by modulating the immune system through regulatory effects rather than cytotoxic ones. It enhances antitumor activity by activating natural killer cells and macrophages, boosting dendritic cells’ antigen-presenting abilities, and inducing apoptosis in regulatory T cells to break immune tolerance. Additionally, it increases MHC class I expression on tumor cells, improving their visibility to the immune system. This approach reshapes the immunosuppressive microenvironment, making it conducive for combination therapies or systemic immune activation. Our study demonstrated that the incorporation of L-TBI significantly augmented the immunological effects elicited by H-BT, thereby facilitating systemic immune responses that resulted in the regression of distal tumors (**Figures 6**,**7**). A recent study investigated the therapeutic effects of combining brachytherapy (BT) and radiotherapy (RT) with dual immune checkpoint inhibition in mice. The authors demonstrated that, when compared to a uniform dose of RT, heterogeneous BT generates a more effective anti-tumor response in distantly unirradiated tumors (23). This suggests that BT demonstrates increased potential when integrated with methodologies that further augment the system’s immunity.

This study has limitations, as it lacks re-challenge experiments and comparisons over time, preventing assessment of whether the observed post-treatment immune activation leads to long-term immune memory or protection. References to “enhancement” of immune parameters are limited to this study’s observation period. Nonetheless, it is the first to show that low-dose whole-body irradiation can induce significant systemic immune activation quickly, paving the way for future research on its long-term effects and mechanisms.

This study represents the inaugural investigation to integrate L-TBI as an immunomodulatory agent in conjunction with H-BT. The combination of L-TBI and H-BT resulted in notable tumor regression in murine models and enhanced immunogenic cell death, as well as the presence of immune cells within the tumor microenvironment and cytokine activity. This novel radiotherapeutic strategy holds potential for managing metastasis in patients with advanced cancer and offers a more convenient and cost-effective alternative to the concurrent use of radiotherapy and immune checkpoint inhibitors.

## Data Availability

The original contributions presented in the study are included in the article/**Supplementary Material**. Further inquiries can be directed to the corresponding authors.
